# Vaccination coverage of patients with type 2 diabetes mellitus: Challenging issues from an outpatient secondary care setting in Greece

**DOI:** 10.3389/fpubh.2022.921243

**Published:** 2022-08-01

**Authors:** Georgios Galanos, Helen Dimitriou, Angelos Pappas, Chrysoula Perdikogianni, Emmanouil K. Symvoulakis, Emmanouil Galanakis, Christos Lionis

**Affiliations:** ^1^Postgraduate Program “Vaccines and Prevention of Infectious Diseases,” School of Medicine, University of Crete, Heraklion, Greece; ^2^Health Center of Arkalohori, 7th Health District of Crete, Crete, Greece; ^3^Laboratory of Child Health, School of Medicine, University of Crete, Heraklion, Greece; ^4^Diabetic Center, Venizeleion General Hospital of Heraklion, Crete, Greece; ^5^Department of Pediatrics, University Hospital, School of Medicine, University of Crete, Heraklion, Greece; ^6^Clinic of Social and Family Medicine, School of Medicine, University of Crete, Heraklion, Greece

**Keywords:** type 2 diabetes, vaccination, infections, prevention, primary care

## Abstract

**Background:**

Increased morbidity/mortality due to vaccine preventable diseases (VPD) is encountered in type 2 diabetes (T2D) people. Aim of this study was to assess their vaccination coverage and describe trends possibly affecting compliance.

**Methods:**

Information on vaccination coverage was retrieved from either documents or interview provided by patients, and/or their vaccination record card at a specialized outpatient diabetes center. The selection of the patients was arbitrary.

**Results:**

An increasing vaccination rate for influenza was observed from 2018 to 2020 among 372 participants. The vaccination coverage for *S.pneumoniae* was 67.2% (PCV13), 20.4% (PPSV23), 26.3% for herpes zoster in individuals ≥60 years, 1.9% for tetanus-diphtheria-pertussis and 1.1% for hepatitis B. A 10.2% of participants were found to be unvaccinated. Vaccination uptake for influenza and PCV13 was related to age, ≥3 comorbidities and long-term follow-up. T2D individuals consecutively vaccinated for influenza were 3.78 times more likely to be also vaccinated with PCV13.

**Conclusions:**

Vaccination rates of patients with T2D show an increasing trend, especially for influenza and *S. pneumoniae*, although the one for *S. pneumoniae* was low. Older people seem more prone to vaccination, the one for herpes zoster was low with infected patients remaining unvaccinated while significantly low coverage was observed for other VPDs. The findings are important to improve effectiveness of preventative services.

## Introduction

Type 2 diabetes (T2D) is increasingly seen with dimensions of a global epidemic due to a rapid increase of new cases according to the International Diabetes Federation (IDF), with their number estimated to reach a rate of one out of ten persons by 2035 (1). Women and men are equally affected, and the onset age is around 45 years. Moreover, the frequency patterns during the last years are increasing among younger individuals. Patients with T2D are particularly vulnerable to a variety of infections such as pneumococcal infections, influenza and shingles (2–4). Special recommendations in the National Immunization Program exist for vulnerable groups of patients with chronic medical conditions including those with T2D (5).

Diabetes represents a significant risk factor for hospitalization among patients with influenza, increasing that for hospital care by 6-fold and Intensive Care admission by 4-fold (6, 7). T2D patients are also at risk of invasive pneumococcal disease (IPD) (8), especially in the older age groups (9). Vaccination recommendations include influenza, *S. pneumoniae*, herpes-zoster, tetanus, diphtheria and pertussis, varicella, measles, rubella and mumps and hepatitis B.

Increased susceptibility of diabetic individuals to hepatitis B is related to the frequent self-blood sampling, nephropathy with external dialysis and potential blood transfusions (10, 11), although a pragmatic risk is expected to be contained due to personal hygiene measures.

Approximately 13% of herpes zoster cases occur in patients with T2D and those at the age range 41–79 years, who show significantly lower cellular immunity to the virus compared to age-matched healthy adults (12). Vaccination with the live attenuated shingles vaccine is reported to be inversely correlated with age, providing 42–70% protection and reduction in terms of incidence and symptoms of postherpetic neuralgia (13).

The population with T2D is also quite prone to tetanus, especially when diagnosed with ulcer in a “diabetic foot,” because the reduced capillary vasculature in the lower extremities-due to peripheral neuropathy and/or peripheral arterial disease-turns frequently into a polymicrobial infection complicated with anaerobic bacteria (14).

In Greece, integrated care is still on debate and comprehensive and person-centered Primary Health Care (PHC) requires a large room for improvement, while the available medical records system is not in use to assess the vaccination coverage of patients in certain vulnerable groups (15, 16). Thus, it was interesting to design a cross-sectional study to look at the vaccination needs of patients with T2D, to explore their status with the ultimate goal to communicate the results to policy makers and healthcare governors.

The aim of this study was to collect information on vaccination coverage of patients with T2D for all vaccines recommended within the National Vaccination Program (NVP) for this clinically vulnerable patient group. Correlations of demographic data, employment status and glycemic control with the vaccination coverage were assessed, in order to describe trends possibly affecting compliance to vaccine recommendations, for future care or local policy use.

## Study population and methods

### Study design

This is a descriptive study, conducted September-December 2020. Information on vaccination coverage of a convenient, consecutive sample of T2D patients, attending the outpatient diabetes care setting of a secondary care hospital, Venizeleion General Hospital of Heraklion, Greece, was collected and analyzed. Variables possibly affecting frequency patterns were tested by using socio-demographic, bio-clinical access and service use data.

### Data collection

The data was collected by the same investigator during a scheduled appointment of the participants at the specialized outpatient diabetes care setting having a long-time expertise and a number of offices were running simultaneously. The approach-selection of the patients was arbitrary.

The inclusion criteria were:

1. Diagnosis of T2D for at least 2 years.
2. Regular follow up at the participant outpatient setting for at least 1 year.
3. Treatment (Tablets or Injectable).
4. Age ≥ 18 years.

Vaccination coverage data were collected through the individual health records of the participants, electronic prescription platform and/or individual health booklet and/or vaccination record card. Vaccination compliance to the National Vaccination Program (5) for adults was investigated. Retrospective data extraction included vaccination coverage information for influenza annually (years 2018, 2019, and 2020), *S. pneumoniae* (PCV13 and PPSV23), herpes zoster-varicella (in adults ≥60 years), hepatitis B, diphtheria, tetanus and pertussis (every 10 years, Tdap / Tdap-IPV or Td) and measles-mumps-rubella (for participants < 50 years old). Moreover, demographic data such as age, gender, residence, period of time under treatment for T2D and type of medication, recent glycosylated hemoglobin level and co-morbidities such as lung / heart disease or nephropathy or malignancy were collected *via* interview, after signed informed consent and by using a form of socio-demographic and clinical information to guide interview.

Correlation of vaccination patterns for T2D recommended vaccines, with years of follow-up, HbA1c, sex, age, place of residence and comorbidities (≥3) was examined. Herpes zoster vaccine with history of shingles, influenza vaccine for the last 3 years and PCV13 in chronic obstructive pulmonary disease /bronchial asthma (COPD/BA) patients, were recorded.

### Statistical analysis

Values are expressed as mean ± standard deviation, unless otherwise stated. Apart from descriptive statistics for vaccine coverage, the binary logistic regression was used for correlations and statistical significance (set at *p* = 0.05) was calculated with the chi-square test (x^2^). The IBM SPSS Statistics 2017 (Version 25.0) software was used for analysis. The study was approved by the Ethics Committee and the Scientific Council of “Venizeleion” General Hospital-Heraklion (Protocol number 44250/2020) and the 7^th^ Health District of Crete (Department of Research and Development Protocol number 58307/2020).

## Results

### Demographics

**Figure 1:**
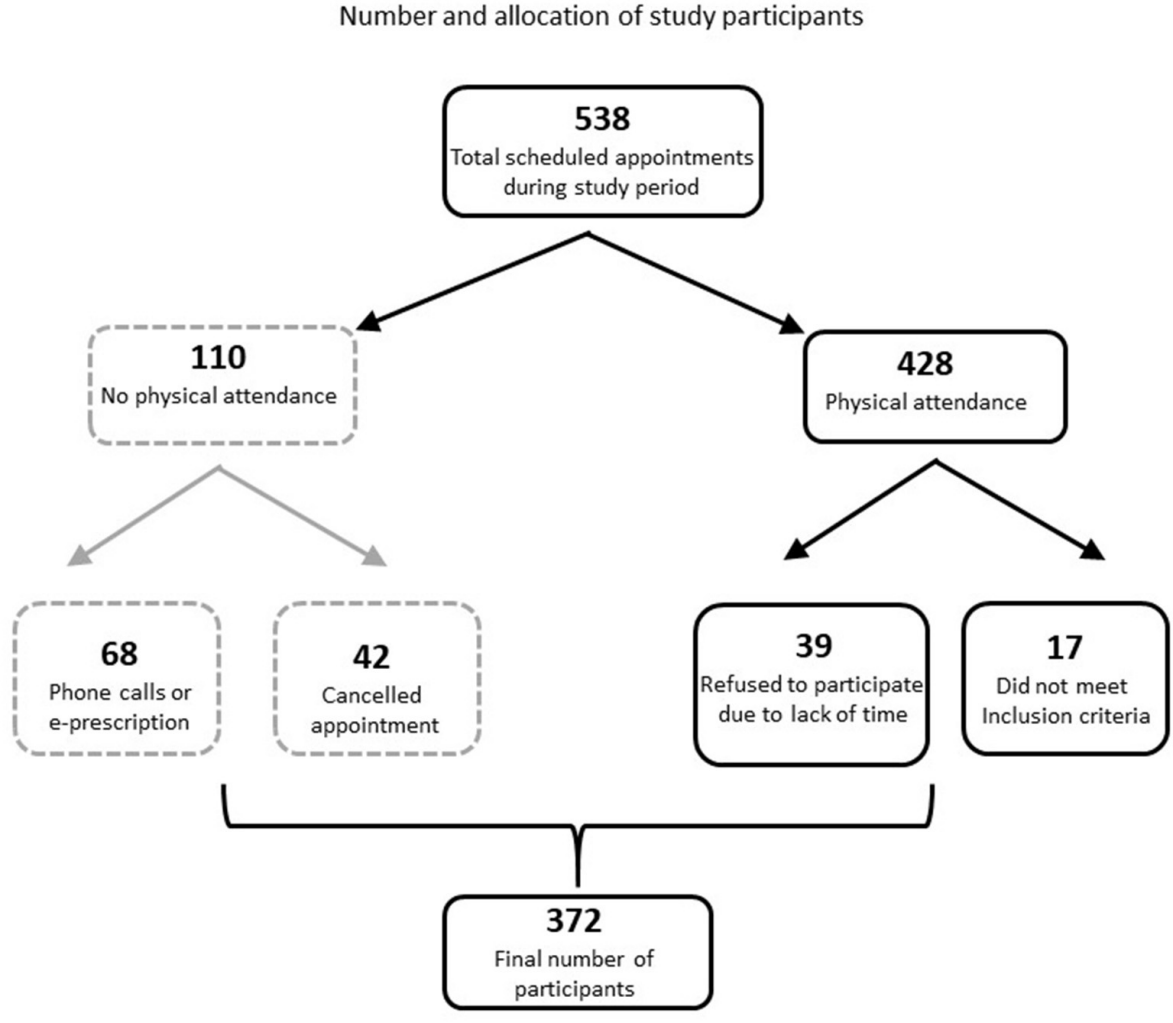
An initial number of 538 appointments had been arranged during the study period. The reasons and numbers of patients of the missing appointments are shown within the dot-line square while the ones for declining participation are shown within the solid-line squares.

The mean age of participants was 68 ± 10.8 years [median 69, min 23-max 92 years] and most (79.6%) were ≥60 years old. The age groups were stratified as: 19–24 yrs: 0.3%, 25–44 yrs: 1.9%, 45–64 yrs: 30.4%, >64 yrs: 67.5%. Males were 53.8%, city inhabitants 52.4% and almost all were covered by social insurance (90.3%). Regarding the employment status of the participants, 51.9% were retired, farmers were 19.6%, private and civil sector employees 4%, 10.2% were long-term unemployed /occasional employees/housewives and of unknown status 14.2%. The flow of participants is depicted in Figure 1.

### T2D related features and comorbidities

The characteristics of diabetes (HbA1c values and applied treatment) as well as the comorbidities of the participants are shown in Table 1. Moreover, the years since diagnosis and on treatment were 12.8 ± 9.5, with a follow-up of 2 to 45 years. A 48.7% of the participants had ≥3 concurrent diseases. Men are more likely to have more co-morbidities, with the exception of asthma (women 82.3%).

**Table 1:** Percentage of T2D individuals with accompanied diseases and T2D related features.

**Feature**	**Frequency**
Dyslipidemia	85.7 %
Hypertension	80.9 %
Heart failure	38.7 %
CAD	20.4 %
COPD	9.7 %
Asthma	4.6 %
Renal disease	2.7 %
Malignancy	7.2 %
OM	62.4 %
Insulin / OM + insulin	30.9 %
IAD + OM	6.7 %
HbA1c % (mean)	7.03 ± 0.9
HbA1c <7.0%	54.3 %
HbA1c > 7.5%	23.9 %

CAD, coronary artery disease; renal disease, end-stage renal disease; OM, oral medication; IAD, injectable antidiabetic drugs (GLP-1); HbA1c, glycosylated hemoglobin.

### Vaccination status against influenza

Vaccination status against seasonal flu for the last three periods is shown in Table 2. Men slightly predominated women in terms of influenza vaccination, in all three studied periods.

**Table 2:** People with T2D vaccinated against seasonal influenza.

	**Total (%)**	**>60 years (%)**	** <60 years (%)**	***p*-value**
2018	57.8	63.4	30.3	<0.0001
2019	73.6	78.7	54	<0.0001
2020	82.5	85.5	71.1	0.003

It is noteworthy that there was a 10.2% who did not have any recorded vaccine in their medical history as adults, mostly men, ≥60 years (63.2%). Twenty more had only got the seasonal flu vaccine the period September-December 2020 (amidst COVID-19 era), predominantly men (70%) and ≥60 years old (65%). Data regarding influenza vaccination of T2D individuals with respiratory and other comorbidities are shown in Table 3. The probability of a T2D individual having ≥3 other chronic diseases to be annually vaccinated against influenza, was 1.97-fold higher in comparison to someone with <3 comorbidities (*p* = 0.001).

**Table 3:** Vaccination for seasonal influenza of T2D individuals *with comorbidities*.

**Comorbidities**	**2020 (%)**	**All 3 periods (%)**
COPD	80	55
Asthma	90	70
Coronary HD	88.1	60
Heart failure	86	60.4
Malignancy	81	55

HD, heart disease; COPD, chronic obstructive pulmonary disease.

### Vaccination status against *S. pneumoniae*

Of all participants, 67.2% had received the conjugate vaccine (PCV13). One third (30.4%) had it within 2020. Only 20.4% were vaccinated against *S. pneumoniae*, with the multivalent polysaccharide vaccine (PPSV-23), 38% of them in 2020. In terms of age, 73.0% of patients ≥65 years had the PCV13 and 55.6% of <65 years. Regarding comorbidities, 72.9% of T2D with ≥3 chronic diseases were vaccinated with PCV13. In particular, individuals with chronic respiratory diseases only at a percentage of 29.4% had PCV13 and all but one was fully vaccinated with both PCV13 and PPSV23. The probability for someone with chronic lung disease (asthma or COPD) to be vaccinated with PCV13 was 2.5 times higher than one without pulmonary disease (*p* = 0.16). Finally, among those eligible for vaccination with both PCV13 and PPSV-23 almost one third (33.8%) have had them.

### Factors affecting influenza and *S. pneumoniae* vaccination

Compliance to influenza vaccine, increases by 3.8 times the probability of PCV13 vaccination (*p* < 0.0001). Patients with comorbidities were more likely to be vaccinated against both influenza and *S. pneumoniae* (PCV13) compared to those without (51.9 vs. 33.5%, *p* < 0.0001). There are other factors affecting compliance such as age (≥ 65 years better vaccinated than youngers, 50.4 vs. 26.6%, *p* < 0.0001), years of follow up (>4 years compared to shorter periods 46.5% vs. 29.5%, *p* < 0.005) and insurance capacity (*p* = 0.003). Other factors such as gender (*p* = 0.057), residence (*p* = 0.221), and Hb1Ac (*p* = 0.158), do not seem to exert any effect on vaccination coverage.

### Vaccination against other infectious diseases

The vaccination rate against shingles (HZV) corresponds to 26.3% although 79.6% were ≥60 years old and 21.4% had it in 2020. Shingles was reported by 42 patients, 35.7% of them had in addition been vaccinated and 88.1% were ≥60 years. No statistical significance was found between vaccinated and naturally infected/non infected individuals (*p* = 0.2). Those born before 1970 were vaccinated for measles-mumps-rubella at a percentage of 86.4%. Regarding the tetanus-diphtheria-pertussis vaccine, a poor 1.9% reported vaccination. Finally, just 1.1% reported vaccination for hepatitis B and, only one individual with chronic kidney disease was adequately vaccinated.

## Discussion

### Main findings

T2D is a chronic condition predisposing to certain infectious diseases which can be prevented by vaccines. Coverage against seasonal influenza at 2018 and 2019 did not reach the minimum target set by the ECDC for European countries (17), while in 2020, rates have exceeded this target. There has been an increasing trend in all age groups for influenza and *S. pneumoniae* vaccines with the ≥60 years' group having the highest coverage This predominance is not unexpected as there is a specific recommendation for them (18). Vaccination for herpes zoster was found low with infected patients remaining unvaccinated. Significantly low vaccination coverage was observed for other VPDs.

### Discussing the study findings under the light of the literature

Individuals with diabetes are 6% more likely to be hospitalized with infections compared to the rest of the population (19). Previous results, from the geographical area of the present study, show that vaccination coverage in an age-matched population with T2D reached 77.7% in 2011 and 73.9% in 2018 (20, 21). Another multicenter study of patients with community-acquired pneumonia reports similar findings, with seasonal influenza vaccination coverage in 2014 reaching 72% (22). A 83.6% of individuals of the same age group with T2D assessed at the national level, was vaccinated for influenza in 2018–2019, a trend also found in the current study conducted in a more restricted geographical area, possibly reflecting a generalized national situation (23). Patients with T2D from primary health care centers and a specialized clinic, had vaccination rates reaching 62.1% for influenza and only 16.1% for *S. pneumoniae* (24), lower than the WHO target for European countries (≥75% for influenza and 65–75% for *S. pneumoniae*) (25, 26). Therefore, fluctuation in the flu coverage throughout the years and differences from one area to another as well as health care facility, exists. Similar trends were reported in US and Great Britain (27, 28). The increasing vaccination for <60 years, from 2018 to 2020, is very encouraging (18). In general, the coverage for influenza has not been satisfying so far, as most European countries do not reach the target with only Great Britain, reaching 72.8% for 2018 and the Netherlands with a rate of 67.8% (29). The improvement achieved during the last year is possibly due to the recent pandemic which raised awareness both of individuals and policy makers.

According to WHO, the mortality rate due to pneumococcal infections is on average 10–20%, and can exceed 50% in high-risk groups (30). T2D individuals are estimated to be almost three times more likely to die from complications associated with bacterial pneumonia (31) and 1.2 times more likely to be hospitalized for pneumonia than the general population. People with diabetes and HbA1c ≥ 9% are 60% more likely to be hospitalized for pneumonia, and even those with HbA1c < 7% have a 22% higher risk compared to the general population (32), making diabetes one of the 3 most common indications for pneumococcal vaccination for people 18–49 years (18). Rates of invasive pneumococcal disease have generally decreased in adults, but those with underlying diseases remain a group at increased risk. Compared to previous data with 54.4% in 2011 and 36.7% in 2018 of the T2D population vaccinated for *S. pneumoniae* (20, 21), the result of the present study (67.2%) is quite encouraging. A multicenter study on community-acquired pneumonia, showed that 44% of T2D patients were vaccinated for *S. pneumoniae*, while in the most recent nationwide study on vaccinations of the elderly, the rates in T2D patients reached 50% for PCV13 and 30.7% for PPSV-23 (22, 23). A low vaccination coverage of 16.1% with the PPSV-23 earlier was attributed to lack of information on the recommendation for the specific vaccine (24), indicating the need for improvement in strategies for vulnerable groups. Nevertheless, in our study, patients with chronic respiratory diseases (asthma or COPD) had not had satisfactory pneumococcal coverage (20% for PPSV23 polysaccharide). Noteworthy, roughly one third of the vaccinated for *S. pneumoniae* had the vaccines within 2020, presumably under the pressure of the COVID-19 threat. Limited knowledge, lack of dissemination and misinformation seem to be the reasons that a large number of participants reported to be unaware of the need for PPSV-23.

Individuals with T2D, especially those 41–79 years, show significantly lower cellular immunity against herpes-zoster virus and approximately 13% of herpes-zoster cases occur in patients with T2D (12). Considering herpes zoster vaccination, 32% of the participants ≥60 years of age were vaccinated, and this is 10% more, compared with that of a recent report assessing vaccination of a convenient sample of patients ≥60 in Greece (23). Unawareness of the indications of the specific vaccine prevails among T2D individuals, especially the oldest of them although two thirds of all herpes-zoster cases are reported at ages>50 years (33). The fact that shingles vaccine was released relatively recently in Greece, could provide an explanation of its rather limited distribution in the general population at the moment but does not fully compensate for the low vaccination rate among T2D people who are regularly visiting a doctor or /and a special center.

As far as tetanus is concerned, a 9-year study in Italy found that 80.2% of cases occurred in people over the age of 65 and the incidence of tetanus in women was three times higher than in men (34). Vaccination against tetanus-diphtheria-pertussis was extremely low in the present study. This carries high risk, as almost 2/3 of the participants are exclusively engaged in agricultural work. The pattern has not changed during the last years since similar results of extremely low rates were reported from others (35). The low tetanus vaccination rate reflects the rate of booster doses recommended every 10 years and this time interval may explain the low rates both in high -risk groups and in the general adult population also reported also in the literature (34, 36).

Hepatitis B vaccination coverage was very low with a coverage rate of 1.07% even in the population with impaired renal function. Only one in 10 reported confirmed vaccination and sufficient level of specific antibodies (anti-HBs). Impaired renal function may lead to extra renal clearance and increased risk of infection due to weakened immune system in combination with frequent hospital visits and use of catheters and needles (11). Hence, the need to vaccinate against hepatitis B patients with diabetes, especially those with impaired renal function, is vital.

Data from different settings and specific age group patients with different chronic diseases vary and caution is required in their interpretation. Nevertheless, similar vaccination rates in patients with different chronic conditions have been noticed (37–39). This could help shaping current health attitudes to influence future care decisions regarding vaccination of patients with chronic diseases.

In search of solutions for non-compliance of patients with chronic conditions (40) to vaccination against VPDs, seemingly a global problem (41–43), better implementation and compliance to vaccination program by attending physicians and nurses are imperative (44). This role was further and highly revealed during the COVID-19 vaccine roll-out (45).

### Impact of the study

T2D patients belong to the high-risk groups for both severe disease and complications from the pathogens reported above. The findings of the present study indicate that the patients may not be adequately informed on the need of extra vaccines. The existence of comorbidities, especially from the lungs, multiplies the vaccination rates against influenza and *S. pneumoniae*. Accurate information on special vaccine recommendations for T2D, preferably by the treating physicians who regularly follow individuals, is desirable. Accessibility is feasible given that recommended vaccines in Greece are provided free of charge in all ages through both private and public services. Although a substantial increase of vaccinated people during 2020 has been recorded, possibly due to the onset of the SARS-CoV-2 virus pandemic, the call for vaccination by the health authorities aiming at shielding the population from infections other than coronavirus (46) seems to be an imperative priority addressed globally (44). Efforts are put forward to increase vaccination coverage in high-risk groups through healthcare professionals' continuing education and increase of public awareness. Primary Health Care physicians have a central role to improve the vaccination coverage in adults' populations and this role is recognized also as important during the pandemic period (47). Noteworthy, in Greece there is no systematic observation of vaccination coverage of adults so far, and this does not facilitate monitoring of vaccination strategies. A better communication between physicians in primary and secondary health care is also visible and an action plan for integrated care for chronic diseases in Greece is still on the health planning agenda (48). Future evidence-based interventions that may have a potential to improve vaccination coverage of general practitioners and indirectly of their patients (49) are imperative.

### Limitations and strengths

Our findings are under the view of some limitations, as the study population was from a single hospital setting which though is the major center for diabetes follow-up in Crete. Retrospective collection has always certain limitations despite the use of multiple sources of information. The impact of pandemic on service use and on decision-making process within the outpatient diabetes care setting, may have slightly altered components of care in regard to data of 2020. However, in terms of captured data in the midst of pandemic, the current study represents a promising set of information for future comparisons. A major advantage is that the interviews were taken by the field researcher himself, giving the opportunity to fully explain the questions to the participants with a homogeneous, compact manner and without interpersonal interview deviations in terms of content and style. The results are indicative of vaccination compliance of people with diabetes who have regular follow up by their doctors.

*Conclusively* vaccination rates of people with T2D show an increasing trend in 2020 especially for seasonal influenza and *S. pneumoniae* although the individuals with a concurrent chronic respiratory disease have still suboptimal vaccination coverage rates for *S. pneumoniae*. Older people seem to be more diligent about vaccination than younger ones. Low vaccination rates were found for herpes zoster with infected patients remaining unvaccinated, albeit the risk of recurrence. Significantly low vaccination coverage is also observed for vaccines against tetanus, diphtheria, pertussis and hepatitis B. Our results provide some evidence on the status of vaccination coverage among T2D people in Crete, Greece and underline the need for initiatives for the improvement of vaccination coverage given their vulnerability to vaccine preventable infections. It also reveals the central role of Primary Health Care in a period where a health care reform is discussed in Greece and especially during the COVID-19 pandemic and the actions needed to be undertaken toward the continuing monitoring of both vaccination coverage assessment and quality improvement programs. A nationwide study is warranted in the field to inform policy makers and plan adequate strategies for prevention at a national level.

## Data availability statement

The original contributions presented in the study are included in the article/supplementary material, further inquiries can be directed to the corresponding author.

## Ethics statement

The studies involving human participants were reviewed and approved by Ethics Committee and the Scientific Council of Venizeleio General Hospital-Heraklion and 7^*th*^ Health District of Crete. The patients/participants provided their written informed consent to participate in this study.

## Author contributions

All authors listed have made a substantial, direct, and intellectual contribution to the work and approved it for publication.

## Conflict of interest

The authors declare that the research was conducted in the absence of any commercial or financial relationships that could be construed as a potential conflict of interest.

## Publisher's note

All claims expressed in this article are solely those of the authors and do not necessarily represent those of their affiliated organizations, or those of the publisher, the editors and the reviewers. Any product that may be evaluated in this article, or claim that may be made by its manufacturer, is not guaranteed or endorsed by the publisher.
