# Erratum: Vaccination coverage of patients with type 2 diabetes mellitus: Challenging issues from an outpatient secondary care setting in Greece

**DOI:** 10.3389/fpubh.2022.1034544

**Published:** 2022-11-10

**Authors:** Frontiers Production Office

**Affiliations:** Frontiers Media SA, Lausanne, Switzerland

**Keywords:** type 2 diabetes, vaccination, infections, prevention, primary care

Due to a production error, the order of affiliations entered incorrectly in the published version, this meant that for authors HD, AP, CP, ES, and CL, affiliations were inaccurate.

In the original version, the author affiliations appeared thus:

Georgios Galanos^1,2^, Helen Dimitriou^1,4*^, Angelos Pappas^5^, Chrysoula Perdikogianni^1,3^, Emmanouil K. Symvoulakis^3^, Emmanouil Galanakis^1,3^ and Christos Lionis^1,6^

^1^Postgraduate Program “Vaccines and Prevention of Infectious Diseases”, School of Medicine, University of Crete, Heraklion, Greece, ^2^Health Center of Arkalohori, 7th Health District of Crete, Crete, Greece, ^3^Laboratory of Child Health, School of Medicine, University of Crete, Heraklion, Greece, ^4^Diabetic Center, Venizeleion General Hospital of Heraklion, Crete, Greece, ^5^Department of Pediatrics, University Hospital, School of Medicine, University of Crete, Heraklion, Greece, ^6^Clinic of Social and Family Medicine, School of Medicine, University of Crete, Heraklion, Greece

The corrected version appears thus:

Georgios Galanos^1,2^, Helen Dimitriou^1,3*^, Angelos Pappas^4^, Chrysoula Perdikogianni^1,5^, Emmanouil K. Symvoulakis^6^, Emmanouil Galanakis^1,5^ and Christos Lionis^1,6^

^1^Postgraduate Program “Vaccines and Prevention of Infectious Diseases”, School of Medicine, University of Crete, Heraklion, Greece, ^2^Health Center of Arkalohori, 7th Health District of Crete, Crete, Greece, ^3^Diabetic Center, Venizeleion General Hospital of Heraklion, Crete, Greece, ^4^Department of Pediatrics, University Hospital, School of Medicine, University of Crete, Heraklion, Greece, ^5^Laboratory of Child Health, School of Medicine, University of Crete, Heraklion, Greece, ^6^Clinic of Social and Family Medicine, School of Medicine, University of Crete, Heraklion, Greece

The publisher apologizes for this mistake. The original version of this article has been updated.

