# Erratum: Vaccination coverage of patients with type 2 diabetes mellitus: Challenging issues from an outpatient secondary care setting in Greece

**DOI:** 10.3389/fpubh.2022.1114452

**Published:** 2023-01-13

**Authors:** Frontiers Production Office

**Affiliations:** Frontiers Media SA, Lausanne, Switzerland

**Keywords:** type 2 diabetes, vaccination, infections, prevention, primary care

Due to a production error, a previously published Erratum (1) introduced errors in the affiliations of four authors. The correct affiliations are as follows.

There was an error regarding the affiliations for Helen Dimitriou. As well as having affiliation 1, they should also have “Laboratory of Child Health, School of Medicine, University of Crete, Heraklion, Greece.”

There was also an error in the affiliation of Angelos Pappas. Instead of “Department of Pediatrics, University Hospital, School of Medicine, University of Crete, Heraklion, Greece,” they should have “Diabetic Center, Venizeleion General Hospital of Heraklion, Crete, Greece.”

There was an error in the affiliations for Chrysoula Perdikogianni and Emmanouil Galanakis. As well as having affiliation 1, they should also have “Department of Pediatrics, University Hospital, School of Medicine, University of Crete, Heraklion, Greece.”

The corrected affiliations are as follows:

Georgios Galanos^1,2^, Helen Dimitriou^1,3*^, Angelos Pappas^4^, Chrysoula Perdikogianni^1,5^, Emmanouil K. Symvoulakis^6^, Emmanouil Galanakis^1,5^ and Christos Lionis^1,6^

^1^ Postgraduate Program “Vaccines and Prevention of Infectious Diseases,” School of Medicine, University of Crete, Heraklion, Greece

^2^ Health Center of Arkalohori, 7th Health District of Crete, Crete, Greece

^3^ Laboratory of Child Health, School of Medicine, University of Crete, Heraklion, Greece

^4^ Diabetic Center, Venizeleion General Hospital of Heraklion, Crete, Greece

^5^ Department of Pediatrics, University Hospital, School of Medicine, University of Crete, Heraklion, Greece

^6^ Clinic of Social and Family Medicine, School of Medicine, University of Crete, Heraklion, Greece

The publisher apologizes for this mistake. The original version of this article has been updated.
